# Hospital Festivals and Meetings

**Published:** 1894-05-26

**Authors:** 


### HOSPITAL FESTIVALS AND MEETINGS.

### CHARING CROSS HOSPITAL FESTIVAL DINNER.

The Charing Cross Hospital held its triennial festival dinner at the Hotel Metropole on Saturday last, and in view of the indebtedness in which the military of the metropolis stand towards the institution, and the interest they display in its welfare, it was not surprising to find this branch of the service well represented. The Duke of Cambridge occupied the chair, and the company, which numbered some 140, included the Duke of Coburg (the president of the hospital), Lord de'Isle, Lieut.-General Sir Reginald Gipps, Alderman Sir E. Inglefield, Lieut.-General R. Bateson, Surgeon-General Sir Guyer Hunter, Sir Joseph Fayrer, Colonel R. Heler Percy, Major Probyn, Captain the Hon. D. Monson, Mr. George J. Drummond and Mr. J. B. Martin (treasurers of the hospital); the Chevalier Harold Smith, Alderman Faudel Phillips, Mr. J. Astley Bloxam, Alderman Frank Green, Mr. H. A. Bosanquet, Mr. A. J. Lawrence, Mr. H. Wagner, Mr. F. G. Hilton Price, Mr. Harman, and Mr. Borrett. Naturally an unendowed hospital which makes but one appeal in the time in which so many others manage to prefer three, must put forward very considerable efforts to achieve a large measure of success, and this was done in the present case. All things considered, fortune has not dealt unkindly in the past with the Charing Cross Hospital. Its previous appeals, which have all been made on behalf of special objects, have in every single case met with a sympathetic response from the public. An accident ward has thus been formed, a medical school of almost unsurpassed excellence has been founded, and, through the munificence of Mr. Passmore Edwards, a convalescent home is about to rise at Limpsfield in Surrey. But affairs are not now so prosperous with the hospital as they were a very few years ago, for reserve funds have been swallowed up and there is a large deficiency on the ordinary current account, the gravity of which the Chairman, who plumed himself on refraining from presenting the usual optimistic after-dinner view of hospital finance, took care to strongly emphasise. Indeed, a better appeal from the chair on behalf of a philanthropic institution has not lately been heard in the Whitehall Rooms. Fortunately, too, it was speedily approached. Royalty proposing the Royal toasts cannot very well avoid being brief, and the Duke of Cambridge could hardly be expected to hold forth at much length on the "Army, Navy, and Reserve Forces." Nor could the speakers who responded to the latter toast be very prolix or pointed, though Alderman Sir E. Inglefield was rather better placed than Lieut.-General Sir R. Gipps, who was very brief, making, nevertheless, an apt contribution by stating from his own experience that the use and privileges of the hospital were thoroughly appreciated by the garrison of London and the soldiers in the metropolis.

Then, in a very chatty and colloquial speech, the Chairman proposed "Prosperity to the Hospital," pointing out that as those gatherings were only triennial they had a right to hope that they would be in result more advantageous than those made annually in connection with similar institutions. The older hospitals and institutions were rather under a disadvantage at the present time with the many new ones which were rising in the metropolis and interesting themselves in the novelties of the day, and which, moreover, had the advantage of being so generally advertised. They might ask why did not the older institutions advertise themselves in the same spirit, but it should be remembered that these had a *locus standi*, that they did their duty, and that they were well established. But at the same time it became needful for those who were interested in the older institutions to exert themselves in order to keep them before the public, not so much in an advertising spirit, but in that useful and necessary spirit which enabled them to keep them going on the principles underlying their establishment. Charing Cross Hospital was in the heart of London, placed in a position of the greatest possible value to the metropolis, and it was only reasonable to hope that there would be sufficient and continued interest shown in it to enable it to go on and prosper. (Cheers.) At the present moment the hospital owed £10,000. He thought it deplorable that such should be the case, and it was their duty in the years to come to keep the institution as efficient as it had been in the past, and to extend its work. (Cheers.) During the three years ending December 31st, 1893, 6,801 in-patients and 60,856 out-patients had benefited by the hospital, and the totals would have been even greater but for the fact that the institution had been necessarily closed for two months of that period in order to improve the sanitary arrangements and bring them up to the requirements of the day. Thus, maintenance on the one hand and improvement on the other had to be provided for, and while the former necessitated no great difference now from the original condition of things, the latter required great outlay. The state of things with regard to their nurses, too, had greatly changed since the establishment of the hospital. They had now to look after them better and provide better accommodation for them, and this required additional expenditure which was not foreseen when the institution was founded. Charing Cross Hospital was not endowed; it lived from hand to mouth, and he asked them to remember that, if the institution did not get the money it required, it would go from bad to worse, and the debt would grow larger still. Excluding legacies—£13,535—which came into the capital account, their income was £27,300, and their expenditure £48,780. "We have no investments," concluded the Duke, "all we can fall back upon is your generosity." (Cheers.)

The Duke of Edinburgh acknowledged the toast, which was most cordially received, his speech being for the most part the reading of a short *resumé* of the position of the institution over which he presided, and which, two days before, he had officially visited. After rehearsing the outcome of the previous appeals already mentioned, his Royal Highness referred to the question of the nursing accommodation, saying that they hoped some philanthropist would come forward and present them with a home for the nurses. They appealed that night, however, for funds to support what he might call the life of the hospital itself. The £6,000 put by in 1888 for bad times no longer existed, and public subscriptions did not make good the balance between fixed income and expenditure. They stood at the present time facing a deficiency of over £11,000, and unless that heavy debt was wiped out they would have to close some of the wards, which meant the bringing of incalculable discomfort on the needy working-classes of the metropolis. During the seventy-four years since its foundation the hospital had relieved 1,000,000 sufferers, and especially was it useful in connection with the street accidents that occurred at times of public rejoicing, there having been, for instance, 198 such cases recorded on the books of the institution during the three days' festivities on the occasion of the Duke of York's marriage. He therefore confidently asked not only those present, but the general public, to respond to his appeal, so that when the next financial statement was issued the incubus of debt might be removed. (Cheers.)

Mr. J. B. Martin proposed the "Medical Staff" in a speech, like so many delivered on occasions of this kind, practically inaudible to numbers in the room, and the compliment was acknowledged by Dr. Black, who specially referred to the difficulty which had been experienced in obtaining a site for a convalescent home, it being the custom with the landowners to step in, when they heard of the overtures being made by the hospital authorities, and buy the land over the latter's heads. The only other toast was "The Chairman," aptly proposed by Sir J. Fayrer, after which subscriptions were announced to the amount of £3,269. The length of the proceedings necessitated the curtailment of a very long musical programme.

### HOSPITAL FOR EPILEPSY AND PARALYSIS.

The annual meeting of the governors and supporters of the Hospital for Epilepsy and Paralysis, Portland Terrace, took place on May 21st. The chair was taken by the Bishop of Marlborough, and there were present the Archdeacon of Middlesex, Dr. Gladstone, F.R S., General Young, Dr. Althaus, Captain Ellis, and several others. The Secretary read the report, which gave the number of in-patients for the year at 114, and of out-patients at 9,084; and stated that the ordinary expenditure of 1893 had been slightly less than that of 1892. In spite of this fact, the expenditure of the hospital would have been greater than the income by more than £200 had it not been for the appropriation for current expenses of legacies, and £100 from the balance with which the year began.

The Chairman, who moved the adoption of the report, said that, considering the bad times and the far greater difficulties of many other hospitals, he did not think that their own deficit was one which need distress them greatly. He was sure the hospital only wanted to be well known to be adequately supported. The inmates were treated with the greatest kindness and attention, and he believed he was right in saying that it was only in such institutions that the poor could obtain the rest and quiet necessary in the treatment of diseases of the nervous system. Further, such hospitals were a valuable factor in the training of medical men, and ought to be supported if only from a feeling of self-interest. In conclusion, he pleaded for an increase of the annual subscriptions.

The Archdeacon of Middlesex, who seconded the motion, said that in giving to an institution we always asked two questions—Is the institution needed? and is it well managed? In this case an affirmative could be given to both questions. The hospital was admirably managed, and the increased quickness and hurry of life was increasing the number of those suffering from nervous diseases.

Among the other speakers were Captain Ellis, who hoped that the committee would see their way to refusing the dona tions of the benefit societies, since their collections and processions being held on Sunday led to "Sabbath breaking"; and Dr. Gladstone, F.R.S., also dwelt on the increase of nervous diseases, especially in those just starting in life. With a vote of thanks to the chairman, the meeting then closed.

